# Personalized reconstruction of large chest wall defect with rapid prototype prosthetic rib: early result and long-term patient outcomes in three cases

**DOI:** 10.1186/1749-8090-10-S1-A156

**Published:** 2015-12-16

**Authors:** Opas Satdhabudha

**Affiliations:** 1 Department of Surgery, Thammasat University Hospital, Pathumthani, 12120, Thailand

## Background/Introduction

The innovation from merging computed tomography (CT) measurement data to rapid prototyping, that now allow the production of solid copies of the patient's bones, has presented thoracic surgeons with new opportunities in large chest wall reconstruction. Since 2010, our department applied this technique to prepare rapid prototype prosthetic ribs (RPPRs) which were customized for each patient.

## Aims/Objectives

We pioneered a personalized chest wall reconstruction with RPPRs in three patients. We then evaluated patient early and long-term outcomes.

## Method

From June 2010 to December 2014, a retrospective review of three patients who underwent resection of the large chest wall tumors and reconstruction with RPPRs was performed. Periodic CT measurements geometric patients' chest contour were used to study.

## Results

Patients, 1 female and 2 males, ranged in age from 48 to 68 years (mean 58 years). All patients underwent resection of the large chest wall tumor. Resection area was extended in anterolateral chest wall (n = 1), and anterior to posterior chest wall (n = 2). Number of resected ribs in each patient was 3, 4 and 5 ribs, respectively. The largest diameter of defects ranged from 15 to 24 centimeters. RPPRs were implanted and muscle flap coverage were used in all cases. No hospital mortality occurred. No patients developed infection of prosthesis. Patient follow-up ranged 12 to 48 months. No paradoxical movements or prosthesis displacement, and no relevant restriction of daily activity occurred in any patients. Geometric chest contour was stable at 6, 12, 24 and 48 months after surgery.

## Discussion/Conclusion

This promising technique results in good patient outcome with functional and stable geometric results. Materials and surgical costs were reasonable, and the rapid prototyping is widely available worldwide. Furthermore, with the 3D model, the thoracic surgeon is able to visualize the surgery accurately beforehand and perform surgery more efficiency.

